# A Pilot Study on Bioactive Constituents and Analgesic Effects of MyrLiq®, a* Commiphora myrrha* Extract with a High Furanodiene Content

**DOI:** 10.1155/2017/3804356

**Published:** 2017-05-24

**Authors:** Antonio Germano, Andrea Occhipinti, Francesca Barbero, Massimo E. Maffei

**Affiliations:** ^1^Farmacia Antoniana, Viale Cesare Balbo 3, 10040 San Gillio, Italy; ^2^Department of Life Sciences and Systems Biology, University of Turin, Via Quarello 15/A, Turin, Italy

## Abstract

The analgesic properties of myrrh* (Commiphora myrrha)* have been known since ancient times and depend on the presence of bioactive sesquiterpenes with furanodiene skeletons. MyrLiq is a* C. myrrha* extract with a standardized content of curzerene, furanoeudesma-1,3-diene, and lindestrene (12.31 ± 0.05 g kg^−1^, 18.84 ± 0.02 g kg^−1^, and 6.23 ± 0.01 g kg^−1^, resp.) and a high total furanodiene content (40.86 ± 0.78 g kg^−1^). A balanced sample of 95 female and 89 male volunteers (with ages ranging from 18 to older than 60 years) exhibiting different pain pathologies, including headache, fever-dependent pain, joint pain, muscle aches, lower back pain, and menstrual cramps, was divided into two groups. The experimental group received 1 capsule/day containing either 200 mg or 400 mg of MyrLiq (corresponding to 8 mg and 16 mg of bioactive furanodienes, resp.) for 20 days, and the placebo group was given the same number of capsules with no MyrLiq. A score was recorded for all volunteers based on their previous experience with prescribed analgesics. For the male volunteers, pain alleviation was obtained with 400 mg of MyrLiq/day for almost all pathologies, whereas, for female volunteers, alleviation of lower back pain and fever-dependent pain was observed with only 200 mg of MyrLiq/day. These results indicate that MyrLiq has significant analgesic properties.

## 1. Introduction

A significant portion of the world population experiences some type of pain, which is one of the major causes of loss of quality of life and medical consultations. Analgesics are among the most prescribed classes of drugs to relieve pain. In addition to disease- or injury-related pain, hyperalgesia (enhanced responses to normally painful stimuli) and allodynia (painful responses to normally nonpainful stimuli) are frequently experienced [1]. The correct use of analgesics includes oral administration, treatment at regular intervals, prescription based on pain intensity, individually adapted dosages, and constant vigilance regarding the necessary information on when and how to administer the medications [2]. In addition to synthetic drugs, several plant extracts are used for their analgesic activity, and approximately 210 plant species belonging to 79 families with activity mediated by opioidergic receptors have been identified in various pharmacological studies [3]. Bioactive natural compounds exerting analgesic activity include alkaloids of opium poppy* (Papaver somniferum)* and cannabinoids of hemp (*Cannabis sativa* var.* indica*) [4]. In addition to monoterpenes from different essential oils [5], other terpenoid classes exhibit analgesic action. For instance, the sesquiterpene parthenolide present in feverfew* (Tanacetum parthenium)* exerts analgesic activity [6, 7], and the sesquiterpene lactone argolide from the aerial part of* Artemisia glabella* exhibits analgesic activity comparable to that of diclofenac [8]. Furanosesquiterpenes with analgesic activity, such as furanoelemanes, furanoeudesmanes, and furanogermacranes, are present in the gum resin extracts of myrrh [9].

Myrrh is the exudate produced by the bark of plants belonging to the genus* Commiphora* (Burseraceae family), which comprises more than 150 species originating mainly from arid tropical and subtropical regions [10, 11]. The so-called true myrrh is produced by* Commiphora myrrha* (Nees) Engl., also known as* C. molmol* Engl. or* Balsamodendron myrrha* Nees. This plant has been used as a wound-healing extract since ancient times, with medicinal uses dating back to Biblical times [12, 13]. The myrrh furanodienes curzerene, furanoeudesma-1,3-diene, and lindestrene are primarily responsible for the myrrh aroma as well as the analgesic activity of myrrh [14].

MyrLiq is a* C. myrrha* extract produced by Biosfered S.r.l. (Italy) and is characterized by a high content of bioactive furanodienes. Here, we report on the chemical analysis of the furanodiene content of MyrLiq and its analgesic action based on a preclinical double-blind controlled study of male and female volunteers.

## 2. Materials and Methods

### 2.1. Reagents

MyrLiq, a myrrh [*Commiphora myrrha *(Nees) Engl.] extract, was provided by Biosfered S.r.l. (Turin, Italy) as a yellowish powder produced from myrrh gum resins with a total furanodiene content > 40 mg/g [15 CMP02-0101-PD01]. The Certificate of Analysis (CoA) of the product is available on the company's web site (http://www.biosfered.com). Sigma-Aldrich (USA) provided the pure standards of* trans*-nerolidol, which were used as internal standards and dissolved in hexane (Sigma-Aldrich, USA) at a final concentration of 10 mg/mL. Aliquots of the stock solutions were stored in 1.5-mL HPLC vials at −80°C until use. The chemical purity and integrity of the standard compound were assessed prior to use.

### 2.2. Identification and Quantification of MyrLiq Furanodienes

One hundred milligrams of MyrLiq-PWD were extracted in a glass tube with 5 mL of acetone : hexane 1 : 1 (VWR International, Radnor, PA, USA) (extraction ratio 1 : 50 w/v) and 500 *μ*g of internal standard (*trans*-nerolidol). The samples were vigorously mixed by vortexing, and the powder was then extracted in an ultrasonic bath at 30°C for 30 min. At the end of the extraction, the samples were mixed by vortexing and centrifuged for 10 min at 5,000*g*. The supernatant of the solvent was collected in a glass tube. The extraction was repeated twice, and the solvent aliquots were combined in the same glass tube.

Prior to analysis, the extracts were loaded into Pasteur pipettes filled with MgSO_4_ (Fluka, USA) to filter the samples and remove any traces of water. The samples were then analyzed by gas chromatography (GC) coupled to mass spectrometry [15] for qualitative analysis of the compounds and by GC coupled to a flame ionization detector (FID) for quantitative analysis. The GC-MS analyses were performed using an Agilent Technologies 6890N gas chromatograph coupled with an Agilent Technologies 5973A mass spectrometer using a Zebron ZB-5MS (Phenomenex, USA) capillary column (30 m length, 250 *μ*m internal diameter, and 0.25 *μ*m film thickness). The injector temperature was set at 250°C, and a constant helium flow (1.0 mL min^−1^) was used as the mobile phase. The following temperature program was used: initial temperature 50°C, followed by a linear thermal gradient of 3°C min^−1^ up to 200°C and a second gradient of 10°C min^−1^ up to 290°C. The final temperature was held for 3 min. The transfer line temperature to the MSD was 280°C, and the ionization energy (*E*_*I*_) was set at 70 eV with a full scan range of 50–300 *m*/*z*. The compounds were identified by comparing their mass spectra to the NIST 98 library using NIST mass spectral search software v2.0.

Quantitative analysis by GC-FID was performed using the same chromatographic parameters described above with the same type of column. The detector (FID) temperature was set at 280°C.

### 2.3. Study Population and Inclusion/Exclusion Criteria

To assess the effects of MyrLiq, we recruited a population of test volunteers (89 men and 95 women) and an identical number of placebo volunteers who were involved in studies performed by Farmacia Antoniana (San Gillio, Italy) under the supervision of medical doctors. Informed consent was obtained. The inclusion criteria encompassed any woman or man between the ages of at least 18 to older than 60 who was experiencing headache, fever-dependent pain, joint pain, muscle aches, lower back pain, or menstrual cramps. The choice of volunteers was completely balanced, and volunteers with known anatomical abnormalities were excluded from this study. Nonspecific symptoms included anorexia, fatigue, reduced mobility, and signs of delirium (e.g., confusion and deterioration of mental or functional status). After explaining the study and obtaining consent, the volunteers were assigned to either the placebo or experimental randomized groups. The randomization was concealed.

### 2.4. MyrLiq Administration and Dosage

The tablets contained either 200 mg or 400 mg of the product (corresponding to 8 mg and 16 mg of bioactive furanodienes, resp.), 395 mg of microcrystalline cellulose, and either 5 mg of magnesium stearate (for the 200 mg capsules) or 195 mg of microcrystalline cellulose and 5 mg of magnesium stearate (for the 400 mg capsules). The choice of the two dosages was established after several trials performed during explorative galenic studies (unpublished). The placebo was indistinguishable in color, taste, and appearance and consisted of all of the elements above except MyrLiq, which was replaced with rice proteins (the additive in MyrLiq). The experimental group received 1 tablet containing either 200 or 400 mg of MyrLiq for 20 days, and the placebo group was given the same number of tablets with no MyrLiq. A score from 0 (representing no effect) to 10 (representing a maximum analgesic effect of MyrLiq) was recorded for all volunteers based on their previous experience with prescribed analgesics (see below). The tablets were administered for 20 days, and, during this time, the volunteers were followed with alternating visits and telephone calls every 2 days. To avoid contamination, the volunteers were asked not to use analgesics or any other natural products for the duration of the study (except the placebo group, in which volunteers were asked to immediately report any symptoms). If symptoms were reported, the volunteers were asked to interrupt the placebo administration and use the analgesics prescribed by their medical doctors. The attending physicians, the outcome assessor, and the statistician were all blinded to the group allocations.

Pathologies included headache, fever-dependent pain, joint pain, muscle aches, lower back pain, and menstrual cramps.

The prescribed analgesics were the following: diclofenac (DI), ketoprofen (KE), ibuprofen (IB), paracetamol (PA), tramadol (TR), and ketorolac (KT).

### 2.5. Statistical Analysis

Kolmogorov–Smirnov tests were used to assess the data distribution. General linear models (GLMs) were used to assess the independent effects of age and sex (random factors) or treatments (fixed factor) on the scores declared by the patients. Nonparametric analysis of variance with pairwise post hoc comparisons was used to assess differences in the scores recorded after treatment with the placebo, 200 mg of MyrLiq, and 400 mg of MyrLiq. The boxplots show the median, quartile, maximum, and minimum score values, and the outliers are identified with open asterisks. Spearman rank correlations between the dose (placebo, 200 mg of MyrLiq and 400 mg of MyrLiq), “score,” and “success” (score > 5) variables were analyzed. Cluster analyses were conducted using the Euclidean distances with the single-linkage method. All statistical analyses were performed using SPSS (v. 22.0, Chicago).

## 3. Results and Discussion

### 3.1. Bioactive Constituents of MyrLiq

The chemical composition of MyrLiq comprises several furanodienes, including the bioactive compounds curzerene, furanoeudesma-1,3-diene, and lindestrene (Table 1). The presence of these three major furanodienes is typical of the genus [13, 16, 17], and these compounds are primarily responsible for the analgesic effects of myrrh extracts [14]. GC-FID quantitative analyses revealed that the total percentage of identified furanodienes was approximately 60% of the total volatile fraction, whereas the total furanodiene content was 40.86 g kg^−1^ (SD = 0.78) (Table 1). These results are in agreement with the typical percentage and content of furanodienes in myrrh reported previously [18, 19].

After the assessment and authentication of the furanodiene content of MyrLiq, we prepared two types of tablets containing either 200 or 400 mg of MyrLiq, corresponding to 8.17 mg and 16.34 mg of total furanodienes, respectively.  Table 2 specifies the content of the individual furanodienes in both tablet formulations.

The tablets and the corresponding placebo, which did not contain MyrLiq, were then administered to the volunteers.

### 3.2. Baseline Characteristics of the Volunteers

The female and male volunteers were selected based on different pain categories, including headache, fever-dependent pain, joint pain, muscle aches, lower back pain, and menstrual cramps. The volunteers were asked to compare the effects of MyrLiq with the drug they had been taking for the specific pain they were experiencing. We asked the volunteers to score the effects of MyrLiq on a scale between 0 and 10, with 0 indicating no effect and 10 indicating an effect comparable to that of the drug they had been using to treat the pain.  Table 3 presents the demographic and baseline characteristics of the volunteers.

In general, the reasons for dropping out of the study in the experimental group included relocation (7), feeling better prior to the end of treatment (8), contrary advice from a family doctor (2), and a family perception of ineffectiveness of MyrLiq (1). The reasons for dropping out in the placebo group included pain (21), contrary advice from a family doctor (3), and a family perception of ineffectiveness of MyrLiq (9). The median follow-up time for both groups was 20 days. The mean tablet intake was 98% (95% CI: 96.5–98.5%) and was similar between the experimental and placebo groups.

Overall, the scores assigned by the patients were explained by the administration of the placebo or MyrLiq (see the results for a single disease), whereas no significant differences related to sex and age were observed (*P* > 0.05 for each model). We considered values ≥ 5 as a threshold score for the volunteers. In general, the level of pain experienced by the volunteers was comparable to the second level of the World Health Organization analgesic ladder [20].

### 3.3. Alleviation of Headache

**Figure 1:**
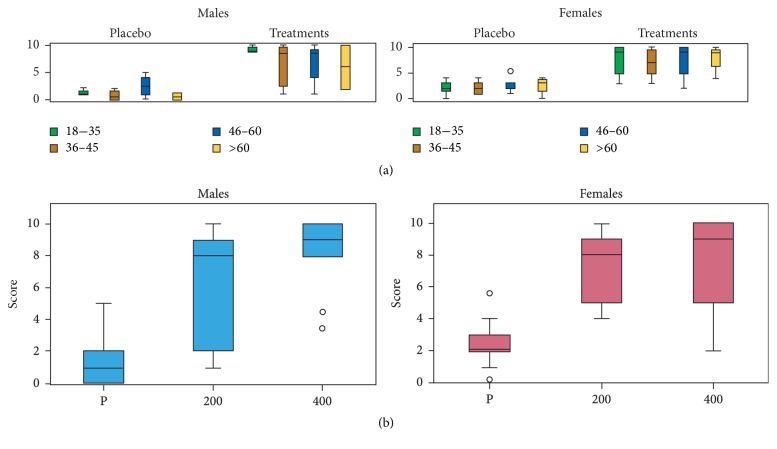
Effects of MyrLiq on headache reduction in male and female volunteers. (a) shows the effects of the treatments and placebo on the different age groups independent of the MyrLiq concentration. (b) shows the effects of the two concentrations of MyrLiq (200 and 400 mg) with respect to the placebo (P) independent of age.

The scores of this group of volunteers were based on comparing the sensations experienced with the treatments to the sensations experienced with KE, IB, and PA. In general, a significant difference was observed between the placebo group and the treatment group (*F*_104,1_ = 133.72, *P* < 0.001). Considering the response of different age groups independent of the concentration of MyrLiq used (Figure 1(a)), male and female volunteers both consistently reacted positively to MyrLiq. The Kruskal Wallis (KW) nonparametric test performed on the three general categories (placebo, 200 mg MyrLiq, and 400 mg MyrLiq) independent of age consistently showed significant differences for both men (KW = 20.396, *N* = 38, *P* < 0.001) and women (KW = 39.102, *N* = 66, *P* < 0.001) (Figure 1(b)). Moreover, the pairwise comparisons between the two concentrations of MyrLiq and the placebo (e.g., 200 mg versus placebo and 400 mg versus placebo) were always significant (*P* < 0.001) for both men and women.

These results indicate that MyrLiq significantly reduces headache pain in both men and women, and the reduction in headache pain was obtained with the lowest concentration of MyrLiq (200 mg). Several plant extracts have been used to alleviate headache pain [21], including cannabis [22], peppermint, and eucalyptus [23, 24]. A recent study of the clinical effects of a nutraceutical preparation based on MyrLiq and ginkgo extracts supplemented with Q10, vitamin B6, and riboflavin over 6 months in patients experiencing headaches reported a significant reduction in headaches [25]. Therefore, our data confirm the efficacy of MyrLiq for headache reduction.

### 3.4. Alleviation of Fever-Dependent Pain

**Figure 2:**
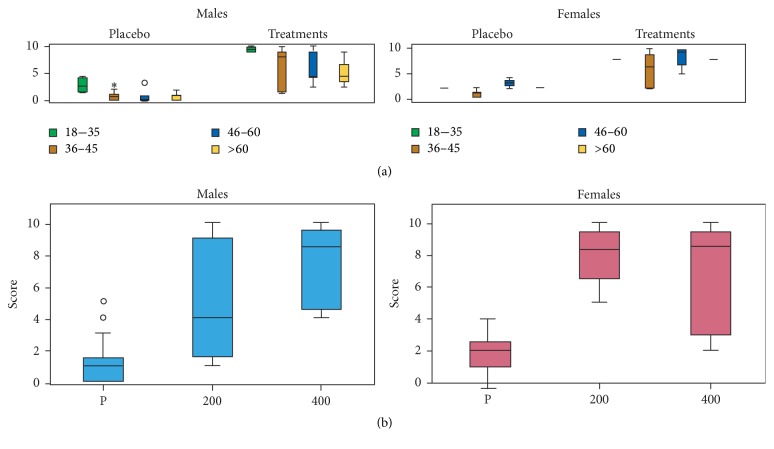
Effects of MyrLiq on fever-dependent pain reduction in male and female volunteers. (a) shows the effects of the treatments and placebo on different age groups independent of the MyrLiq concentration. Outliers are reported as open circles and far outliers (extreme values) as asterisks. (b) shows the effects of the two MyrLiq concentrations (200 and 400 mg) compared with the placebo (P) independent of age.

The scores of this group of volunteers were based on a comparison of the sensations experienced with the treatments to the sensations experienced with PA. In general, significant differences were observed between the placebo group and the treatment group (*F*_62,1_ = 65.22, *P* < 0.001) for both female and male volunteers, and these differences were independent of the concentration of MyrLiq used (Figure 2(a)). The KW tests, which were independent of age, consistently showed significant differences for both men (KW = 19.930, *N* = 38, *P* < 0.001) and women (KW = 13.170, *N* = 24, *P* < 0.001) (Figure 2(b)). The pairwise comparisons between the two MyrLiq concentrations and the placebo were always significant (*P* < 0.001) for both men and women.

Pain caused by fever was already effectively reduced by 200 mg of MyrLiq in both the male and female volunteers, with a marked effect in women (the only group able to reach a threshold > 5). MyrLiq did not lower the fever (i.e., had no antipyretic activity). However, it did reduce the side effects of fever, including headache, general muscular pain, and dizziness.

### 3.5. Alleviation of Joint Pain

**Figure 3:**
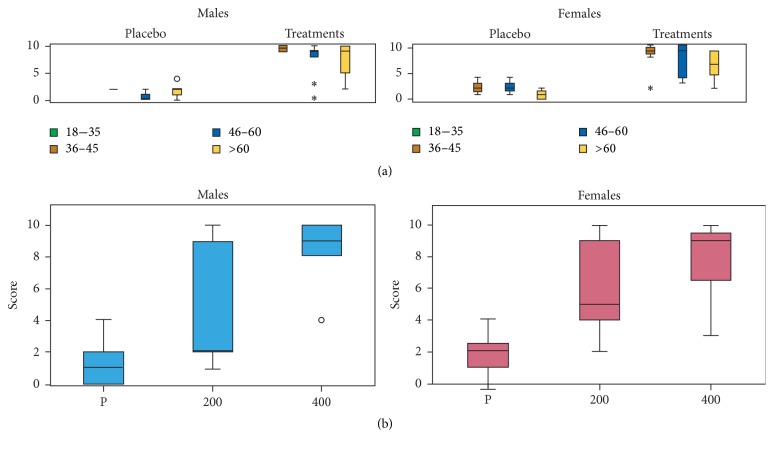
Effects of MyrLiq on joint pain reduction in male and female volunteers. (a) shows the effects of the treatments and placebo on different age groups independent of the MyrLiq concentration. Outliers are reported as open circles and far outliers (extreme values) as asterisks. (b) shows the effects of the two MyrLiq concentrations (200 and 400 mg) with respect to the placebo (P) independent of age.

The scores of this group of volunteers were based on comparing the sensations experienced with the treatments to the sensations experienced with DI and KE. In general, a significant difference was observed between the placebo group and the treatment group (*F*_86,1_ = 161.58, *P* < 0.001) for both the female and male volunteers independent of the concentration of MyrLiq used (Figure 3(a)). The KW test consistently showed significant differences for both men (KW = 26.133, *N* = 40, *P* < 0.001) and women (KW = 29.786, *N* = 46, *P* < 0.001) (Figure 3(b)), independent of age. For men, a significant difference (*P* < 0.001) was observed only when comparing the placebo and 400 mg of MyrLiq, whereas the pairwise comparisons between the two MyrLiq concentrations and the placebo were always significant (*P* < 0.001) for women.

Joint pain is often associated with osteoarthritis [26] and includes pathologies affecting articular cartilage, subchondral bone, synovium, ligaments, and periarticular muscles [27]. Notably, MyrLiq was more effective for women than men when used at a dose of 200 mg. The latter group reported significant effects only at a dose 400 mg of MyrLiq. There are accepted and assumed biological differences between women and men, including differences in pain thresholds and analgesic responses to pain medications [15]. Meta-analysis studies have observed that women report higher pain severity at lower thresholds and have lower tolerance of noxious stimulation than men [28]. Increasing evidence indicates that nutraceutical-based combinations of chondroprotective and/or anti-inflammatory components can effectively reduce joint pain without measurable side effects. MyrLiq at a dose of 100 mg kg^−1^ exhibited prominent analgesic activity, with an inhibition rate of 70.57%, and significantly reduced joint pain and stiffness in subjects with mild osteoarthritis [29]. Therefore, our results confirm the analgesic effects of MyrLiq on joint pain. With regard to other plant extracts, sesquiterpenes from* Aquilaria* spp. and extracts from plants of the genus* Celastrus* were also found to reduce joint pain [30, 31].

### 3.6. Alleviation of Muscle Aches

**Figure 4:**
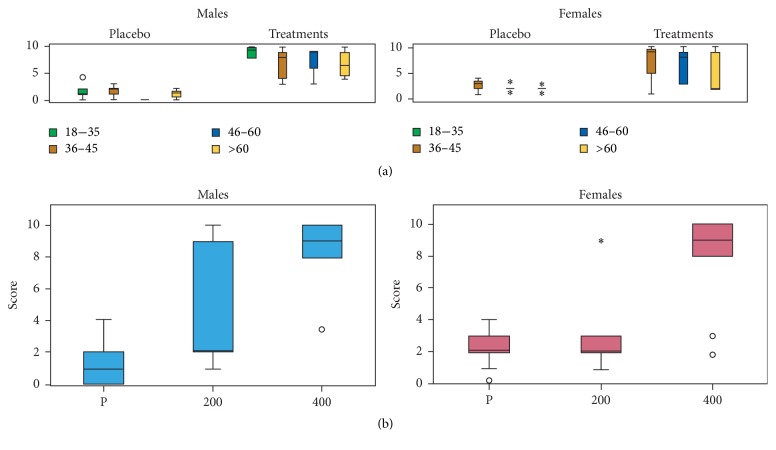
Effects of MyrLiq on the reduction of muscle aches in male and female volunteers. (a) shows the effects of the treatments and placebo on different age groups independent of the MyrLiq concentration. Outliers are reported as open circles and far outliers (extreme values) as asterisks. (b) shows the effects of the two MyrLiq concentrations (200 and 400 mg) with respect to the placebo (P) independent of age.

The scores of this group of volunteers were based on comparing the sensations experienced with the treatments to the sensations experienced with DI, KE, and PA. In general, a significant difference was observed between the placebo group and the treatment group (*F*_72,1_ = 100.799, *P* < 0.001) for both female and male volunteers, independent of the concentration of MyrLiq used (Figure 4(a)). The KW test consistently showed significant differences in both men (KW = 32.733, *N* = 44, *P* < 0.001) and women (KW = 11.487, *N* = 28, *P* < 0.001) (Figure 4(b)), independent of age. For both women and men, a significant difference (*P* < 0.001) was only observed when comparing the placebo and 400 mg of MyrLiq.

Muscle aches, along with joint pain, are among the most prevalent and distressing symptoms, particularly in diseased populations [32, 33] or after intense physical activity [34]. Our results indicate that this symptom was alleviated when MyrLiq was used at dose of 400 mg. However, the effects of lower concentrations were not significant. Reports on the ability of other plants to alleviate muscle aches include a study describing the effects of the monoterpene camphor from* Cinnamomum camphora* [35].

### 3.7. Alleviation of Lower Back Pain

**Figure 5:**
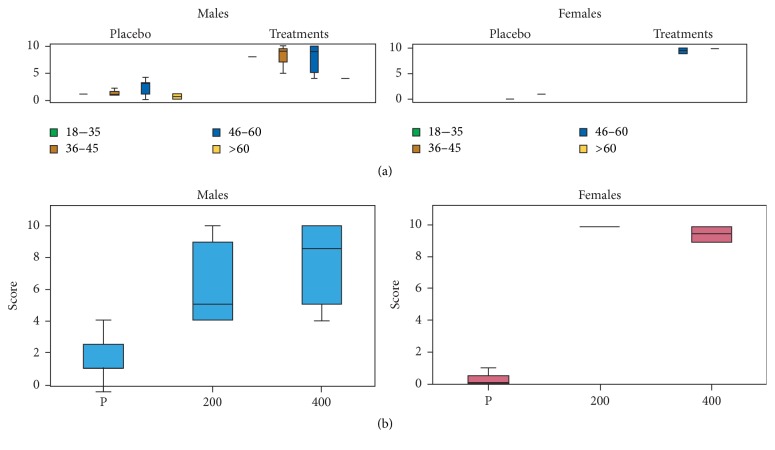
Effects of MyrLiq on lower back pain reduction in male and female volunteers. (a) shows the effects of the treatments and placebo on different age groups independent of the MyrLiq concentration. (b) shows the effects of the two MyrLiq doses (200 and 400 mg) with respect to the placebo (P) independent of age.

The scores of this group of volunteers were based on comparing the sensations experienced with the treatments to the sensations experienced with DI, KE, TR, and KT. In general, a significant difference was observed between the placebo group and the treatment group (*F*_28,1_ = 65.964, *P* < 0.001) for both female and male volunteers independent of the concentration of MyrLiq used (Figure 5(a)). The KW test consistently showed significant differences that were independent of age in men (KW = 15.505, *N* = 22, *P* < 0.001) but not in women (KW = 4.205, *N* = 6, *P* > 0.05) due to a lack of reports (dropouts) for the 200 mg dose (Figure 5(b)). However, the differences between the women receiving 400 mg and placebo were significant (*P* = 0.043). In men, a significant difference (*P* < 0.001) was observed when the placebo was compared with either the 200 mg or 400 mg dose of MyrLiq.

Lower back pain (LBP) generally has a favorable outcome, with significant improvement within 4 weeks [36]. However, LBP imposes a substantial economic burden on people living in industrialized societies [37]. A significant reduction of LBP in men was observed after administration of 200 mg and 400 mg of MyrLiq. For female volunteers, we could only evaluate the positive effects with the 400 mg dose of MyrLiq because the sample population receiving 200 mg was not sufficient to obtain a statistical comparison with the placebo treatment due to dropouts. A recent study described the role of plant extracts in the reduction of LBP, with particular reference to preparations based on* Capsicum frutescens* cream or plaster [37],* Harpagophytum procumbens*, and* Salix alba* extracts [38].

### 3.8. Alleviation of Menstrual Cramps

**Figure 6:**
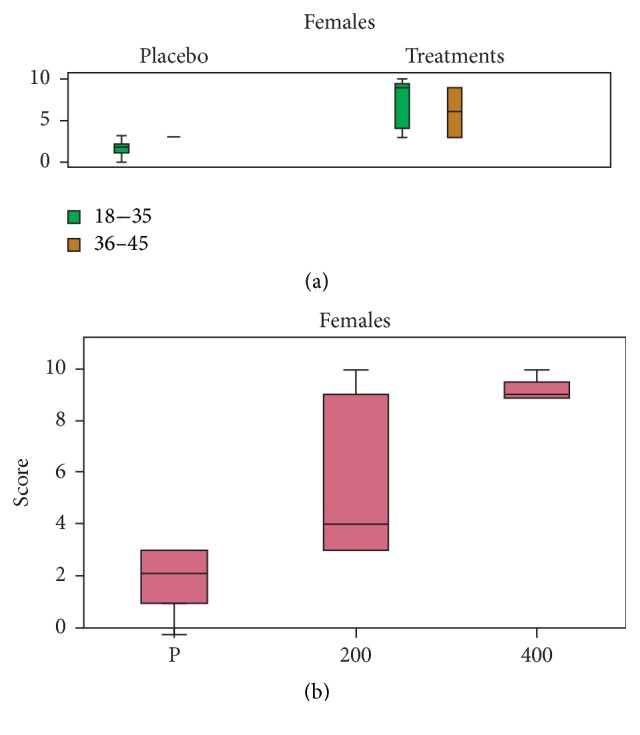
Effects of MyrLiq on the reduction of menstrual cramps in female volunteers. (a) shows the effects of the treatments and placebo on different age groups independent of the MyrLiq concentration. (b) shows the effects of the two MyrLiq concentrations (200 and 400 mg) with respect to the placebo (P) independent of age.

The scores of this group of volunteers were based on comparing the sensations experienced with the treatments to the sensations experienced with KE and IB. In general, a significant difference was observed between the placebo group and the treatment group (*F*_20,1_ = 24.569, *P* < 0.001) in the female volunteers independent of the concentration of MyrLiq used (Figure 6(a)). The KW test showed significant differences (KW = 13.840, *N* = 20, *P* < 0.001) (Figure 6(b)) independent of age. The differences between the placebo and both the 200 mg and 400 mg doses of MyrLiq were consistently significant.

Primary dysmenorrhea refers to painful menstrual cramps without an organic cause. Although severe menstrual problems are rarely reported, this problem is associated with increased physical symptoms and depression [39]. MyrLiq reduced pain from menstrual cramps, with significant effects already observable at the 200 mg dose of MyrLiq. However, the score was below the limit threshold (i.e., 5). Other plants have been shown to reduce menstrual cramps and disorders. For instance,* Mentha *×* piperita* preparations can apparently reduce the severity of primary dysmenorrhea via certain analgesic mechanisms [40].* Phyllanthus muellerianus* extracts are also traditionally used to treat menstrual disorders [41], and* Wedelia trilobata* is effective against menstrual pain and reproductive problems in women [42].

### 3.9. Cluster Analysis of the Responses of Men and Women to MyrLiq

To evaluate the correlations between the two MyrLiq concentrations and the placebo in both males and female volunteers, we performed cluster analyses (CA) using the Euclidean distances and single-linkage method.

**Figure 7:**
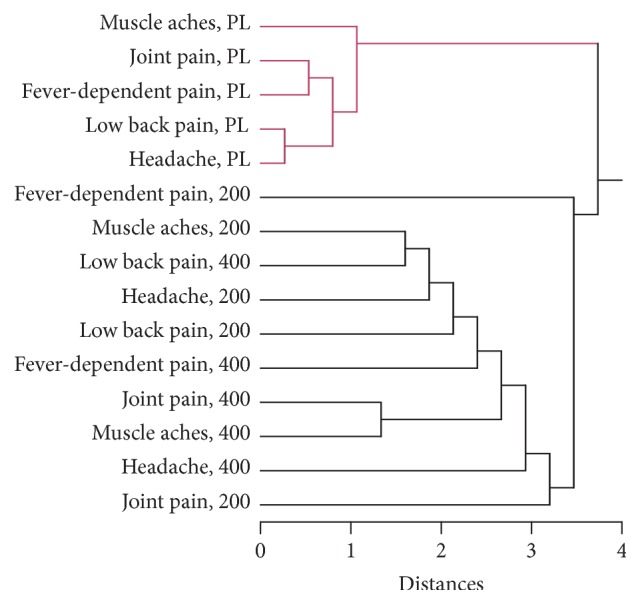
Cluster analysis of the analgesic responses of male volunteers after the administration of placebo, 200 mg of MyrLiq, and 400 mg of MyrLiq. The cluster analysis was performed using the single-linkage method, and the Euclidean distances are indicated. PL = placebo.

In the male volunteers (Figure 7), CA revealed the presence of two main clusters, the first containing all of the placebo results and the second including all of the treatments. In the latter, the responses to fever-dependent pain after treatment with 200 mg of MyrLiq showed low statistical linkage with the other treatments, whereas a close statistical linkage was observed between the responses to joint pain and muscle ache relief after treatment with 400 mg of MyrLiq. This subcluster was linked to the responses to fever-dependent pain and headache after treatment with 400 mg of MyrLiq. The results of this analysis revealed that there was a difference between the placebo and the treatments and emphasized the higher efficiency of the 400 mg dose of MyrLiq in male volunteers for the treatment of most of the pathologies studied.

**Figure 8:**
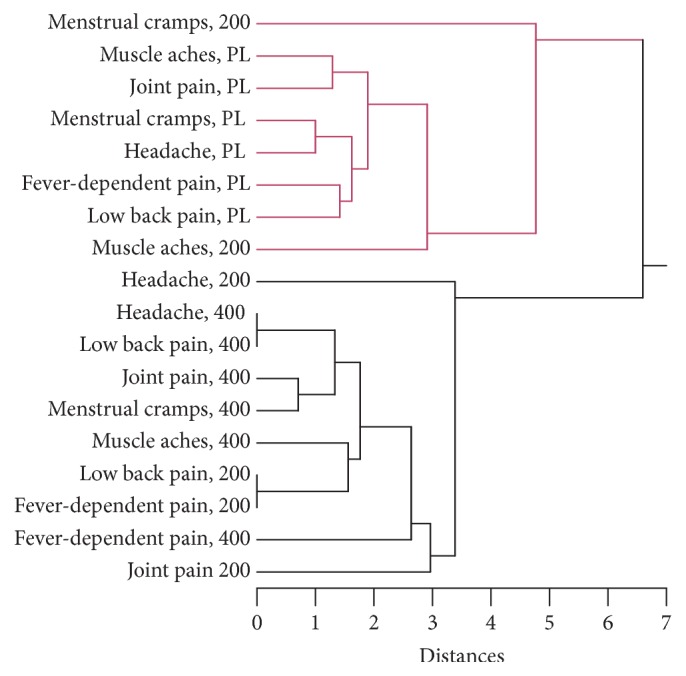
Cluster analysis of the analgesic responses of female volunteers after the administration of placebo, 200 mg of MyrLiq, and 400 mg of MyrLiq. Cluster analysis was performed using the single-linkage method, and the Euclidean distances are indicated. PL = placebo.

In female volunteers, CA revealed the presence of two main clusters (Figure 8). In the first cluster, all placebo results were linked to the responses of the volunteers with menstrual cramps and muscle aches after administration of 200 mg of MyrLiq. This result confirms the low efficacy of 200 mg of MyrLiq for these pathologies. In the second cluster, a low statistical linkage was observed between the alleviation of headache and joint pain with respect to the other treatments after administration of 200 mg of MyrLiq. However, a close statistical linkage was observed between the responses of the volunteers with headaches, LBP, joint pain, and menstrual pain after administration of 400 mg of MyrLiq. The alleviation of LBP and fever-dependent pain showed a close statistical linkage after administration of 200 mg of MyrLiq. Overall, these results indicate that MyrLiq is effective at a dose of 200 mg for pathologies such as LBP and fever-dependent pain, whereas, for the remaining pathologies, the best results were obtained with the 400 mg dose.

## 4. Conclusions

MyrLiq is a myrrh extract with a high content of bioactive furanodienes. The results of this study indicate that MyrLiq has analgesic activities against some of the most prevalent and distressing pain symptoms, particularly headaches, muscle aches, joint pain, lower back pain, fever-dependent pain, and menstrual cramps. A direct comparison with some of the most frequently used drugs (e.g., diclofenac, ketoprofen, ibuprofen, paracetamol, tramadol, and ketorolac) revealed that MyrLiq has similar effects, although it required a longer course of treatment (20 days). In male volunteers, the effects were particularly significant at a dose of 400 mg of MyrLiq/day for almost all pathologies, whereas, for female volunteers, LBP and fever-dependent pain were already alleviated after treatment with 200 mg of MyrLiq/day. No side effects were reported by any of the volunteers. Our results confirm the analgesic properties of myrrh furanodienes [14, 43, 44] and support their application as a natural remedy for a wide range of pathologies in which analgesic effects are required to alleviate pain and improve quality of life.

## Figures and Tables

**Table 1:** Furanodiene content of MyrLiq. Data are expressed as the relative area percentage and content (standard deviation).

Compound	Furanodiene area percentage	Content (g kg^−1^)
Curzerene	17.93 (0.20)	12.31 (0.05)
Furanoeudesma-1,3-diene	27.44 (0.17)	18.84 (0.02)
Lindestrene	9.08 (0.06)	6.23 (0.01)
Unknown furanodiene (MW = 216)	0.44 (0.01)	0.30 (0.004)
Dihydrolinderalactone	1.91 (0.02)	1.31 (0.02)
Acetoxy-furanodiene (MW = 274)	0.87 (0.05)	0.59 (0.03)
Acetoxy-furanodiene (MW = 232)	1.85 (0.04)	1.27 (0.03)
Total	59.51 (0.43)	40.86 (0.78)

**Table 2:** Content of furanodienes in the two types of tablets containing either 200 mg or 400 mg of MyrLiq.

Compound	Furanodiene content in tablets containing 200 mg of MyrLiq (mg)	Furanodiene content in tablets containing 400 mg of MyrLiq (mg)
Curzerene	2.46	4.92
Furanoeudesma-1,3-diene	3.77	7.54
Lindestrene	1.25	2.50
Unknown furanodiene (MW = 216)	0.06	0.12
Dihydrolinderalactone	0.26	0.52
Acetoxy-furanodiene (MW = 274)	0.12	0.24
Acetoxy-furanodiene (MW = 232)	0.25	0.50
Total	8.17	16.34

**Table 3:** Baseline characteristics of the volunteers.

Demographics	Experimental group, MyrLiq administration	Placebo group
Number of volunteers	184	184
Number of women (%)	95 (51.6)	95 (51.6)
Number of men (%)	89 (48.4)	89 (48.4)
Median age (range)	38 (19–61)	38 (19–63)
Age range		
18–35	21 (W), 9 (M)	21 (W), 9 (M)
36–45	27 (W), 26 (M)	27 (W), 26 (M)
46–60	29 (W), 30 (M)	29 (W), 30 (M)
Over 60	18 (W), 24 (M)	18 (W), 24 (M)
MyrLiq-PWD, 200 mg	34 (W), 39 (M)	34 (W), 39 (M)
MyrLiq-PWD, 400 mg	61 (W), 50 (M)	61 (W), 50 (M)

*Baseline level*		
Number for headaches	33 (W), 17 (M)	33 (W), 17 (M)
Comparison with KE, IB, PA		
Number for fever-dependent pain	12 (W), 19 (M)	12 (W), 19 (M)
Comparison with PA		
Number for joint pain	23 (W), 20 (M)	23 (W), 20 (M)
Comparison with DI, KE		
Number for muscle aches	14 (W), 22 (M)	14 (W), 22 (M)
Comparison with DI, KE, PA		
Number for lower back pain	3 (W), 11 (M)	3 (W), 11 (M)
Comparison with DI, KE, TR, KT		
Number for menstrual cramps	10 (W)	10 (W)
Comparison with KE, IB		
Number of tablets (days)	1 (20)	1 (20)
Volunteers not completing the study (%)	18 (9.9)	33 (18.2)
Women not completing the study (%)	11 (11.4)	23 (24.1)
Men not completing the study (%)	7 (8.3)	10 (11.8)

*Notes*. M = men; W = women; DI = diclofenac; KE = ketoprofen; IB = ibuprofen; PA = paracetamol; TR = tramadol; KT = ketorolac.
